# Citrulline and kynurenine to tryptophan ratio: potential EED (environmental enteric dysfunction) biomarkers in acute watery diarrhea among children in Bangladesh

**DOI:** 10.1038/s41598-023-28114-0

**Published:** 2023-01-25

**Authors:** Rina Das, Md. Ahshanul Haque, Rukaeya Amin Sobi, Al-Afroza Sultana, Murad Alam Khan, Amran Gazi, Mustafa Mahfuz, Baitun Nahar, Pradip Kumar Bardhan, Tahmeed Ahmed

**Affiliations:** 1 Nutrition and Clinical Services Division, icddr,b, 68 Shaheed Tajuddin Ahmed Sharani, Dhaka, 1212 Bangladesh; 2 Department of Environmental Health Sciences, Rollins School of Public Health, Emory University, Atlanta, GA 30322 USA; 3 Faculty of Medicine and Life Sciences, University of Tampere, 33100 Tampere, Finland; 4 James P. Grant School of Public Health, BRAC University, Dhaka, 1212 Bangladesh; 5 Department of Global Health, University of Washington, Seattle, WA 98104 USA

**Keywords:** Environmental sciences, Biomarkers, Diseases, Health care, Medical research

## Abstract

Two emerging biomarkers of environmental enteric dysfunction (EED) include plasma citrulline (CIT), and the kynurenine (KYN): tryptophan (TRP)/ (KT) ratio. We sought to investigate the plasma concentration of CIT and KT ratio among the children having dehydrating diarrhea and examine associations between concentrations of CIT and KT ratio with concurrent factors. For this analysis, we used cross-sectional data from a total of 102, 6–36 months old male children who suffered from non-cholera acute watery diarrhea and had some dehydration admitted to an urban diarrheal hospital, in Bangladesh. CIT, TRP, and KYN concentrations were determined at enrollment from plasma samples using ELIZA. At enrollment, the mean plasma CIT concentration was 864.48 ± 388.55 µmol/L. The mean plasma kynurenine, tryptophan concentrations, and the KT ratio (× 1000) were 6.93 ± 3.08 µmol/L, 33.44 ± 16.39 µmol/L, and 12.12 ± 18.10, respectively. With increasing child age, KYN concentration decreased (coefficient: − 0.26; 95%CI: − 0.49, − 0.04; *p* = 0.021); with increasing lymphocyte count, CIT concentration decreased (coef.: − 0.01; 95% CI: − 0.02,0.001, *p* = 0.004); the wasted child had decreased KT ratio (coef.: − 0.6; 95% CI: − 1.18, − 0.02; *p* = 0.042) after adjusting for potential covariates. The CIT concentration was associated with blood neutrophils (coef.: 0.02; 95% CI: 0.01, 0.03; *p* < 0.001), lymphocytes (coef.: − 0.02; 95% CI: − 0.03, − 0.02; *p* < 0.001) and monocyte (coef.: 0.06; 95% CI: 0.01, 0.11; *p* = 0.021); KYN concentration was negatively associated with basophil (coef.: − 0.62; 95% CI: − 1.23, − 0.01; *p* = 0.048) after adjusting for age. In addition, total stool output (gm) increased (coef.: 793.84; 95% CI: 187.16, 1400.52; *p* = 0.011) and also increased duration of hospital stay (hour) (coef.: 22.89; 95% CI: 10.24, 35.54; *p* = 0.001) with increasing CIT concentration. The morphological changes associated with EED may increase the risk of enteric infection and diarrheal disease among children. Further research is critically needed to better understand the complex mechanisms by which EED biomarkers may impact susceptibility to dehydrating diarrhea in children.

## Introduction

Although oral rehydration therapy has greatly reduced diarrhea-associated mortality, the burden of diarrheal disease persists in children below 5 years who are living under unsanitary conditions and limited public health resources. This contributes to 43% of stunted growth and impaired cognitive development, affecting one-fifth of children worldwide and one-third of children in low- and middle-income countries (LMICs)^1,2^.About 80% of the deaths from diarrhea among children happen in the African and South-East Asian regions including Bangladesh. According to the United Nations Children's Emergency Fund (UNICEF), the mortality rate among children younger than age 5 years in Bangladesh has declined from 143 to 30 per 1000 live births but the trends in the prevalence of childhood diarrhea remained mostly unchanged during 1990–2018^3^. Even with the progress in the management of diarrhea, it is still responsible for a death toll of about 525,000 deaths per year in under-five children worldwide^4^. However, Bangladesh remains among the top 15 countries with a high prevalence of childhood mortality attributable to ARI (Acute respiratory infection) and diarrhoeal disease^5^. The vicious cycle of enteric infection and malnutrition often leads to enteropathy for extended periods in young children when adequate water and sanitation are lacking^1,6^. This type of enteropathy is known as Environmental Enteric Dysfunction (EED). EED, previously known as tropical enteropathy or environmental enteropathy, is a sub-acute inflammatory condition of the small intestinal mucosa of unknown etiology^7^. It is characterized by structural changes in the small intestine including villous atrophy and crypt hyperplasia compromising nutrient absorption and pathogenic barrier (increased permeability and inflammatory cell), impaired gut immune function, malabsorption, growth faltering, and generally asymptomatic, as distinct from the diarrheal disease^6,8^.

Numerous biomarkers have been tested to gauge various elements of the proposed EED pathway because EED is a complicated condition with an uncertain origin and unclear diagnostic criteria. Five categories—intestinal injury and repair, permeability and absorption, microbial translocation, intestinal inflammation, and systemic inflammation—were used to group the putative biomarkers in a recent review by Harper et al.^9^. Plasma citrulline (CIT), a sign of intestinal injury and repair, and the kynurenine: tryptophan (KT) ratio, a marker of systemic inflammation, are two newly discovered biomarkers. It is possible to measure intestinal epithelial cell loss, enterocyte bulk, and absorptive activity using CIT, a nonessential amino acid that is largely generated by enterocytes^10^. A brand-new biomarker of the systemic immune response is the KT ratio. Pro-inflammatory cytokines, such as tumor necrosis factor-alpha (TNF-alpha), activate the indoleamine 2,3-dioxygenase (IDO1) enzyme during inflammation, which increases tryptophan catabolism to kynurenine and causes low tryptophan and high kynurenine concentrations as well as a raised KT ratio^11^. Low tryptophan concentrations have been linked to slower linear growth rate, and tryptophan availability for protein synthesis is reduced^12,13^. Additionally, kynurenine itself may have immunomodulatory properties, such as lowering T cell survival and proliferation^14^.

One animal study reported lower plasma CIT levels in neonatal calves with diarrhea compared to healthy control^15^. Tryptophan (TRP), a plant-derived essential amino acid (EAA) is needed to support growth and health in humans. Low plasma TRP, high KYN, and elevated KT ratio are found to be associated with infections (e.g. patients with inflammatory bowel disease suffer from diarrhea) and chronic immune activation^2^. In diseases such as sepsis, type 2 diabetes, obesity, inflammatory bowel disease, and immunodeficiency syndrome, the KT ratio has been utilized as a measure of systemic inflammation^16–20^. Additionally, it was discovered that among children in Malawi, serotonin/TRP and KT ratios were favorably linked with intestinal permeability^21^. Since tryptophan cannot be converted back into protein after being converted to KYN or serotonin, their ratios to TRP can be utilized as a proxy indicator of inflammatory conditions. Plasma citrulline might be a good parameter for mucosal barrier injury in pediatric patients^22^. In animals with parvoviral enteritis, the plasma CIT concentration has been studied to show acute small intestinal injury and assess its prognostic importance^23^. Studies looked at whether citrulline levels in kids receiving a myeloablative allogeneic transplant regimen correspond with clinical indicators of intestinal damage^24^. An animal study found plasma citrulline is a sensitive safety biomarker for small intestinal injury in rats^25^.

Each year, there are over 1.7 billion instances of diarrhea worldwide, making it the second largest cause of death for children under five^26^. Acute infectious diarrheal diseases are caused by several microbial pathogens. The bacterial pathogens include *Vibrio Cholerae,* Enterotoxigenic *E. coli, Shigella, Salmonella*, and *Campylobacter*. Rotavirus is the most prominent viral diarrheagenic pathogen and also the leading cause of infantile diarrhea, whereas *Entamoeba histolytica, Giardia lamblia, and Cryptosporidium* are the important parasites causing diarrhea^27^. Based on the pathogenetic mechanisms, these organisms may be broadly divided into two groups—secretory and invasive^28^. There is insignificant inflammation or structural change in the intestinal mucosa and the diarrhea is watery^29^. On the other hand, *Shigella* (the prototype invasive pathogen) invades the gut mucosa and induces an inflammatory reaction, producing a dysenteric illness^30^. In developing countries, recent studies show that the commonest attributable microbial causes of moderate-to-severe diarrheal illnesses in infants and young children are*: rotavirus, Cryptosporidium*, and Enterotoxigenic *E. coli* producing heat-stable toxins^31^. Long-term exposure to enteric pathogens causes structural changes in the gut, including inflammation, epithelium destruction, blunting of the intestinal villa, and decreased nutritional absorption^32–35^. While the morphological changes associated with EED may increase susceptibility to invasive enteric pathogens, no studies to date have examined the impact of childhood diarrhea on EED biomarkers.

Data are limited from LMICs including Bangladesh on inflammatory and pathological changes in the gut wall among children with dehydrating diarrhea. Although EED is recognized as an important predictor of susceptibility to diarrheal disease, no research to date has examined the mechanisms by which EED may cause diarrheal disease^36^. In this study, the primary objectives were to (1) examine associations between baseline concentrations of these EED biomarkers and concurrent factors; and (2) hypothesize whether the raised EED biomarkers have any role in the hospital outcome of diarrheal children.

## Result

### Characteristics of the participants

A total of 102 venous blood samples were analyzed for citrulline, kynurenine, and tryptophan concentrations. The mean age at enrollment was 12.24 ± 4.78 months. The mean duration of diarrhea before hospitalization was 24.02 ± 10.86 h. More than 80% of patients had vomiting ≥ 3 times/day and 23.5% of patients were febrile. About 75% of children were breastfed. Around 85% of participants’ family income was more than 10,000 taka per month. 96% of children were vaccinated for the EPI (Extended Program on Immunization) vaccines according to their age. Children generally had a lower prevalence of stunting (13.73%), wasting (11.76%), and underweight (13.73%) at the time of enrollment. At baseline, the mean plasma CIT concentration was 864.48 ± 388.55 µmol/L. The mean plasma kynurenine and tryptophan concentrations and the KT ratio (× 1000) were 6.93 ± 3.08 µmol/L, 33.44 ± 16.39 µmol/L, and 12.12 ± 18.10, respectively. Around 76% of children living in a house had cemented floor material, 82% had improved toilet facility in the household, only 28% of children’s caregivers' used boil water as a water treatment method for drinking purposes, and more than 90% of caregivers used soap and water for their handwashing practice and around 80% caregivers washed hand with soap before feeding a child. The mean duration of hospital stay was 54.84 ± 33.97 hours and the mean total stool output was 2158.12 ± 1590.9 gm. And 97.06% of enrolled children were discharged after recovering from diarrhea (Table 1).

**Table 1:** Baseline socio-demographic, anthropometric, clinical characteristics, and laboratory findings of the enrolled male children (6–36 months) * (n* = *102).*

Variables	n = 102 (%)
Age (months)*	12.24 ± 4.78
Duration of diarrhea before hospitalization (hours)*	24.02 ± 10.86
Breastfeed	77 (75.49)
Number of members in the household*	4.51 ± 1.44
Maternal education	
Below primary	15.69 (16)
Primary and above	86 (84.31)
Family income (BDT) > = 10,000/ month	86 (84.31)
Anthropometry	
Stunted	14 (13.73)
Wasted	12 (11.76)
Underweight	14 (13.73)
Outcome	
Total stool output from randomization to diarrhea resolved (gm)*	2158.12 ± 1590.9
Duration of hospital stay (hour)*	54.84 ± 33.97
Outcome	
Left against medical advice	1 (0.98)
Transferred to another ward	2 (1.96)
Usual discharge	99 (97.06)
Baseline laboratory investigations	
Gut biomarkers*	
Citrulline (µmol/L) (*n* = *101)*	864.48 ± 388.55
Kynurenine (µmol/L) (*n* = *102)*	6.93 ± 3.08
Tryptophan (µmol/L) (*n* = *102)*	33.44 ± 16.39
KT ratio (ratio × 1,000) (*n* = *102)*	12.12 ± 18.10
Complete blood picture*	
Hemoglobin (Hb) gm/dl	10.18 ± 1.5
Total WBC count (10^9^/L)	11.48 ± 4.33
Neutrophils (%)	52.3 ± 14.86
Eosinophils (%)	0.98 ± 2.02
Basophils (%)	0.24 ± 0.14
Lymphocytes (%)	40.47 ± 14.38
Monocytes (%)	5.99 ± 2.07
Blood biochemistry*	
Serum sodium (mmol/L)	136.72 ± 4.43
S. potassium (mmol/L)	4.27 ± 0.56
S. chloride (mmol/L)	109.18 ± 5.03
S. TCO2 (mmol/L)	15.9 ± 2.85
S. creatinine (micromole/L)	27.65 ± 6.33
Blood glucose (mmol/L)	5.25 ± 0.84
Pathogen isolated from stool	
*Cryptosporidium*	5 (4.90)
*V. cholerae*	3 (2.94)
EPEC	1 (0.98)
EAEC	50 (49.02)
ETEC	1 (0.98)
*Shigella*	1 (0.98)
*Campylobacter*	2 (1.96)
Rotavirus antigen (positive)	80 (68.0)
Household characteristics	
Floor material	
Cemented	68 (76.4)
non-cemented	21 (23.6)
Available improved toilet facility	73 (82.02)
Handwashing practice	
Water only	6 (6.74)
Soap and water	83 (93.26)
Use of water treatment method	
Boiled	25 (28.09)
not boiled	64 (71.91)
Wash hands with soap before feeding a child	71 (79.78)

*mean ± SD (standard deviation); TCO_2_: total carbon dioxide; Stunting: height/length for age z score < -2; Wasting: weight for height z score < -2; Underweight: weight for age z score < -2; EAEC: Enteroaggresive *E. coli;* EPEC: Enteropathogenic *E. coli*; ETEC: Enterotoxigenic *E. coli.*

Baseline citrulline, kynurenine, and tryptophan concentrations were positively correlated with one another; tryptophan correlated negatively with kynurenine, and the KT ratio correlated negatively with tryptophan, and positively with kynurenine which was statistically significant (Table 2).

**Table 2:** The correlation coefficient for gut health biomarkers at enrollment.

	Citrulline	Kynurenine	Tryptophan	KT ratio
Citrulline	1	0.1687	0.2139*	0.8452
Kynurenine		1	− 0.5836*	0.8618*
Tryptophan			1	− 0.8999*
KT ratio				1

*KT* kynurenine: tryptophan.

*P value < 0.05.

### Biomarkers of EED at baseline and associated predictors

With increasing child age, kynurenine concentration decreased (coef. − 0.26; 95% CI: − 0.49, − 0.04; *p* = 0.021); increasing lymphocyte count, CIT concentration decreased (coef. − 0.01; 95% CI: − 0.02,0.001, p = 0.004); wasted child had lower KT ratio (coef. − 0.6; 95% CI: − 1.18, − 0.02; *p* = 0.042) after adjusting for potential covariates. However, there were no other associations between markers of intestinal injury repair and systemic inflammation and breastfeeding status, maternal education, WASH (water, sanitation, and hygiene), and isolated pathogens (EAEC and rotavirus) from baseline fecal samples (Table 3).

**Table 3:** Gut health biomarkers at baseline and associated predictors.

Variables	Citrulline (µmol/L)	*P* value	Kynurenine (µmol/L)	*P* value	Tryptophan (µmol/L)	*P* value	KT ratio (ratio × 1,000)	*P* value
	Adjusted Coef. (95% CI)*		Adjusted Coef. (95% CI)*		Adjusted Coef. (95% CI)*		Adjusted Coef. (95% CI)*	
Age (months)	− 0.1 (− 0.32,0.11)	0.346	** − 0.26 (− 0.49, − 0.04)**	**0.021**	− 0.2 (− 0.44,0.05)	0.113	0.13 (− 0.26,0.53)	0.507
Breastfeed	− 0.03 (− 0.27,0.2)	0.773	− 0.07 (− 0.31,0.17)	0.583	− 0.18 (− 0.45,0.08)	0.177	0.3 (− 0.13,0.73)	0.170
Wasting	0.07 (− 0.25,0.38)	0.678	0.03 (− 0.29,0.36)	0.840	0.32 (− 0.04,0.68)	0.084	** − 0.6 (− 1.18, − 0.02)**	**0.042**
Lymphocyte count	** − 0.01 (− 0.02,0)**	**0.004**	0.01 (− 0.01,0.01)	0.435	0.003 (− 0.01,0.01)	0.548	0 (− 0.02,0.01)	0.757
Maternal education (primary and above)	0.19 (− 0.14,0.51)	0.254	0.08 (− 0.25,0.41)	0.640	0.17 (− 0.19,0.54)	0.350	− 0.27 (− 0.86,0.32)	0.367
Improved toilet facility	− 0.15 (− 0.43,0.13)	0.292	− 0.28 (− 0.57,0.01)	0.055	− 0.09 (− 0.41,0.22)	0.558	− 0.09 (− 0.6,0.42)	0.714
Use boiled water for drinking	0.04 (− 0.2,0.28)	0.739	− 0.05 (− 0.29,0.19)	0.689	− 0.06 (− 0.33,0.21)	0.646	0.08 (− 0.36,0.51)	0.729
EAEC	0.06 (− 0.15,0.27)	0.572	− 0.11 (− 0.32,0.11)	0.335	0.04 (− 0.2,0.28)	0.723	− 0.19 (− 0.58,0.2)	0.326
Rotavirus	0.11 (− 0.15,0.37)	0.398	− 0.07 (− 0.33,0.2)	0.620	− 0.12 (− 0.41,0.18)	0.430	0.17 (− 0.3,0.64)	0.481

Coef.: coefficient; 95% CI: 95% confidence interval; Models adjusted for all the variables mentioned in the table at enrollment. Values represent the beta coefficient and 95% CI from linear regression models. For continuous predictors, this represents the change in outcome corresponding to a one-unit increase in the predictor. For dichotomous predictors, this corresponds to the change in mean concentration from the reference group.

In Table 4, after adjustment of age in separate linear regression models the tryptophan concentration and the KT ratio were not associated with blood leukocyte concentrations (lymphocytes, monocytes, neutrophils, eosinophils, and basophils) as a marker of systemic inflammation (Table 4). However, CIT concentration was associated with neutrophils (coef.: 0.02; 95% CI: 0.01, 0.03; *p* < 0.001), lymphocytes (coef.: − 0.02; 95% CI: − 0.03, − 0.02; *p* < 0.001) and monocyte (coef.: 0.06; 95% CI: 0.01, 0.11; *p* = 0.021); kynurenine concentration was negatively associated with basophil (coef.: − 0.62; 95% CI: − 1.23, − 0.01; *p* = 0.048).

**Table 4:** Concurrent predictors associated with citrulline, kynurenine, and tryptophan concentrations and the KT ratio at baseline with blood leukocyte concentrations (lymphocytes, monocytes, neutrophils, eosinophils, and basophils) as a marker of systemic inflammation.

Variables	Citrulline (µmol/L)		Kynurenine (µmol/L)		Tryptophan (µmol/L)		KT (ratio × 1000)	
	Adjusted Coef. (95% CI)*	*p*-value	Adjusted Coef. (95% CI)*	*p*-value	Adjusted Coef. (95% CI)*	*p*-value	Adjusted Coef. (95% CI)*	*p*-value
Total WBC count x10^9^L	− 0.02 (− 0.04, 0.01)	0.151	0.01 (− 0.01, 0.02)	0.617	0.01 (− 0.02, 0.03)	0.454	− 0.01 (− 0.06, 0.03)	0.530
Neutrophils	**0.01 (0.01, 0.02)**	** < 0.001**	0.002 (− 0.003, 0.01)	0.356	0.002 (− 0.01, 0.01)	0.526	− 0.002 (− 0.04, 0.01)	0.759
Eosinophil	− 0.01 (− 0.05, 0.04)	0.812	− 0.01 (− 0.05, 0.03)	0.571	− 0.004 (− 0.06, 0.05)	0.864	− 0.002 (− 0.10, 0.09)	0.952
Basophil	− 0.42 (− 1.15, 0.31)	0.255	** − 0.62 (− 1.23, − 0.01)**	**0.048**	− 0.66 (− 1.44, 0.12)	0.096	0.70 (− 0.68, 2.09)	0.315
Lymphocytes	** − 0.02 (− 0.02, − 0.01)**	** < 0.001**	− 0.002 (− 0.01, 0.003)	0.437	− 0.002 (− 0.01, 0.01)	0.504	0.002 (− 0.01, 0.02)	0.681
Monocyte	**0.06 (0.01, 0.10)**	**0.018**	− 0.01 (− 0.05, 0.03)	0.592	0.01 (− 0.04, 0.06)	0.679	− 0.03 (− 0.13, 0.06)	0.475

Coef.: coefficient; 95% CI: 95% confidence interval; *Models adjusted for age at enrollment. Values represent the beta coefficient and 95% CI from linear regression models. For continuous predictors, this represents the change in outcome corresponding to a one-unit increase in the predictor.

The co-pathogens (EAEC and rotavirus) isolated from baseline stool samples were not associated with citrulline, kynurenine, and tryptophan concentrations and the KT ratio at baseline (Table 5).

**Table 5:** Association of citrulline, kynurenine, and tryptophan concentrations and the KT ratio at baseline with isolated pathogens in stool culture.

Co-pathogens isolated	Citrulline (µmol/L) *		Kynurenine (µmol/L) *		Tryptophan (µmol/L) *		KT (ratio × 1,000) *	
	Adjusted Coef. (95% CI)*	*p*-value	Adjusted Coef. (95% CI) *	*p*-value	Adjusted Coef. (95% CI). *	*p*-value	Adjusted Coef. (95% CI)*	*p*-value
EAEC	0.03 (− 0.17,0.23)	0.780	− 0.05 (− 0.22,0.12)	0.531	0.1 (− 0.11,0.32)	0.341	− 0.27 (− 0.65, 0.12)	0.170
Rotavirus	0.12 (− 0.12,0.36)	0.341	− 0.14 (− 0.38,0.09)	0.232	− 0.18 (− 0.44,0.08)	0.179	0.22 (− 0.22, 0.65)	0.328

Coef.: coefficient; 95% CI: 95% confidence interval; *Models adjusted for age at enrollment. Values represent the beta coefficient and 95% CI from linear regression models.

### Biomarkers of EED at baseline and disease outcome

The EED biomarkers: kynurenine, tryptophan, and KT ratio had no association with the duration of hospitalization and total stool output. But with increasing CIT concentration, total stool output (gm) increased (coef.: 793.84; 95% CI: 187.16, 1400.52; *p* = 0.011) and also increased duration of hospital stay (hour) (coef.: 22.89; 95% CI: 10.24, 35.54; *p* = 0.001) and both were statistically significant (Table 6).

**Table 6:** Association of citrulline, kynurenine, and tryptophan concentrations and the KT ratio at baseline with diarrhea outcome.

Gut EED biomarkers	Total stool output (gm)		Duration of hospital stay (hour)	
	Adjusted Coef. (95% CI)*	*p*-value	Adjusted Coef. (95% CI)*	*p*-value
Citrulline (µmol/L)	**793.84 (187.16, 1400.52)**	**0.011**	**22.89 (10.24, 35.54)**	**0.001**
Kynurenine (µmol/L)	− 21.06 (− 778.62, 736.50)	0.956	− .04 (− 16.08, 16.01)	0.996
Tryptophan (µmol/L)	− 509.69 (− 1092.10, 72.73)	0.086	− 6.62 (− 19.17, 5.92)	0.297
KT (ratio × 1,000)	328.13 (− 3.03, 659.29)	0.052	4.49 (− 2.81, 11.79)	0.225

Coef.: coefficient; 95% CI: 95% confidence interval; Separate models adjusted for age at enrollment. Values represent the beta coefficient and 95% CI from linear regression models.

## Discussion

Comparisons to the body of existing literature are challenging since this study is the first to explore the effects of dehydrating diarrhea on CIT and KT ratio as potential novel indicators of alterations in systemic inflammation and EED.

Age, lymphocyte count (a measure of systemic inflammation and immunological function), and wasting were the three primary groups into which concomitant covariates that were reliably related with citrulline, kynurenine, and tryptophan concentrations and the KT ratio tended to cluster^37,38^. In our study we also found that with increasing child age, kynurenine concentration decreased, increasing lymphocyte count, and CIT concentration decreased. Some evidence suggested that tryptophan and citrulline were positively correlated with household wealth (e.g., socioeconomic status index and the presence of domestic animals in a household)^39^. But in our study, we could not observe any association with the presence of domestic animal and citrulline or kynurenine, as most of the enrolled children were from urban site. Citrulline, kynurenine, and tryptophan concentrations and the KT ratio, on the other hand, were only weakly significantly correlated with markers of anthropometry, IYCF (Infant and child feeding) practices, and maternal features, as other research suggests^12,40,41^, but not all^12,42^ of the previous literature. There were only weak correlations in the current study between the KT ratio, citrulline, kynurenine, and tryptophan concentrations, and the indicators of water, sanitation, and hygiene (WASH). Recent community-based randomized controlled efficacy trials of three WASH interventions have been performed. These studies were predicated on the idea that fecal–oral pollution in the presence of subpar WASH causes EED^43–46^. In Bangladesh, WASH interventions reduced intestinal permeability and inflammation in infants as young as 3 months old when compared to controls, but they were later linked to higher EED biomarkers in infants as old as 28 months^47–49^. However, none of the three trials found a correlation between the WASH interventions and children's growth, suggesting the interventions may not have been sufficiently intense to have a significant influence^43–46^.

In the present study, citrulline was negatively associated with concurrent concentrations of lymphocytes and the Kynurenine was negatively associated with basophil count. The relationship between intestinal injury and repair and systemic inflammation was further supported by the favorable associations of citrulline with neutrophil and monocyte count^9^. However, somewhat unexpectedly, we did not observe a significant association between blood leukocyte concentrations (as immune biomarkers) and tryptophan, or the KT ratio. Decreased citrulline and kynurenine levels have been reported during infections in animal models^13^, likely due to increased IDO1 activity during inflammation^50^. Higher plasma kynurenine levels could very well be associated with better maintenance of T-cell homeostasis in healthy individuals, although such data have not, to our knowledge, been reported previously. This is why the negative correlation between citrulline and kynurenine levels and lymphocyte and basophil count observed in the current study was not entirely unexpected. A study conducted among rural Laotian children found, that the KT ratio was not associated with blood leukocyte concentrations (lymphocytes, monocytes, neutrophils, eosinophils, and basophils) but tryptophan level is positively associated with Lymphocyte count^51^. Confirmation of this hypothesis would require further work with more specific characterization of systemic inflammation.

We did not find any association of pathogens (EAEC and rotavirus) isolated from the fecal samples of our study children. Similar findings were observed among the children in Zambia, the EED severity score was significantly higher among asymptomatic controls compared to cases with rotavirus diarrhea (*p* = 0.02)^26^. Another study analyzed a total of 16 age-paired stool samples: 8 diarrheal samples positive for one diarrhoeagenic *E. coli* pathotype and 8 stool samples from healthy children and they observed almost similar findings: arginine levels were similar in both groups, but citrulline levels were higher in healthy samples^52^. However, impacts from the metabolism of citrulline or tryptophan may also play a role in important pathways. These essential metabolites might be less readily available in children due to changed host or microbial metabolism or signaling, higher needs, or both. We also found that lower plasma tryptophan levels are not surprisingly associated with biomarkers of barrier disruption and intestinal and systemic inflammation^53^. This information might be useful to identify mechanisms and signaling molecules involved in the crosstalk between EED biomarkers and the isolation of diarrhoeagenic *E. coli* and other pathogens in the stool.

In patients with inflammatory bowel illnesses, HIV patients, and those who are critically ill, plasma citrulline, which is virtually solely produced by the enterocytes, is a marker of enterocyte mass^54^. It could be a quantitative biomarker of small intestine mass integrity in a group with tropical enteropathy and HIV-associated villous atrophy in Zambia that correlates with crypt depth and xylose absorption^55^. In our study, we found almost 68% of children had rotaviral diarrhea. Viral infections damage small bowel enterocytes and cause low-grade fever and watery diarrhea without blood^56^. Plasma Citrulline levels reflect enterocyte mass and children with viral watery diarrhea have acute enterocyte volume loss due to damage small bowel enterocytes. However, its application in the context of children with dehydrating diarrhea is yet to be explored. In our analysis, we found that raised citrulline levels increased stool output among dehydrated hospitalized children and subsequently caused prolonged hospital stays. This might be due to the children who had higher citrulline levels had increased school output and had worse hospital outcomes due to more acute intestinal damage in dehydrating diarrhea. From a systematic review and meta-analysis^57^, citrulline levels are strongly negatively correlated with intestinal disease severity with regard to enteropathies (coeliac disease, tropical enteropathy, mucositis, acute rejection in intestinal transplantation, but not Crohn's disease), and strongly positively correlated with small bowel length in patients with short bowel syndrome. Citrulline levels were 10 μmol/L lower compared to controls, and citrulline cut-off levels have an overall sensitivity and specificity of 80%. According to these findings, citrulline may be a sign of acute intestine damage or intestinal insufficiency^57^. The importance of plasma citrulline as a biomarker of gut mass, where plasma citrulline levels are predictive of intestinal healing, has also been highlighted by studies in children with short bowel syndrome^58,59^. Overall, further studies are recommended as exploratory EED biomarkers among children with dehydrating diarrhea which is essential to foster the hypothesis and to understand completely the clinical relevance of citrulline among the diarrhea patients for better hospital outcome.

This analysis has several limitations. A primary limitation of this study is the lack of clear diagnostic criteria for EED. We used cross-sectional data and had a small sample size; therefore, we are unable to draw any conclusions about the causality of the link between EED biomarkers and dehydration acute diarrhea. Based on univariate relationships with the outcome of diarrheal disease, data-driven selection of factors to include in adjusted models may be more susceptible to unmeasured confounding. Moreover, we did not analyze fecal markers of intestinal inflammation in the trial for our cost constraints. As the data were collected from hospital-admitted male children of an urban hospital in Bangladesh, results may not be generalizable for all. Nevertheless, the present study provided new insights into the role of CIT and KT ratios with dehydrating diarrhea and their hospital outcome.

We provide the first estimates, to our knowledge, of the association between dehydrating watery diarrhea and EED biomarkers. Our findings not only identify the predictors of EED biomarkers among children having diarrhea but also stress the urgent requirement for EED measures to be included in upcoming research projects in order to better understand the intricate correlations between diarrheal disease and EED biomarkers. Future research examining the association between EED biomarkers and diarrheal disease should therefore utilize longitudinal follow-ups.

## Methodology

### Study design, study setting, and study population

The current analyses are based on data from a subsample of 102 participants enrolled in the VS002A clinical trial^60^. The VS002A trial is a randomized, double-blind, two-cell superiority clinical trial comparing WHO-ORS and amino acid-based ORS: VS002A conducted in children, presenting with non-bloody acute non-cholera diarrhea with some dehydration in the Dhaka Hospital of icddr,b situated in Dhaka, capital of Bangladesh. The Dhaka Hospital is the largest diarrheal disease hospital in the world. An average of 250 patients are treated in the hospital each day. Details about the study site have been reported elsewhere^61^.

In brief, the study was conducted among 312 male (to facilitate separate collection of urine and stool) children who were 6–36 months old presenting with non-cholera acute watery diarrhea (onset ≤ 48 h) and some dehydration, admitted to Dhaka Hospital of icddr,b in between June 2021 and September 2022.

Children were eligible to participate in the study if they were 6–36 months, duration of diarrhea ≤ 48 h, some dehydration (judged clinically according to the “Dhaka method”)^62^, and written informed consent was obtained by either parent/guardian. Children were excluded from the study if they presented with any of the following: Severe malnutrition (Weight-for-length WLZ/weight-for-height WHZ/ weight-for-age WAZ < − 3 or presence of nutritional edema), patients with cholera, bloody diarrhea, presence of systemic illness (e.g. Pneumonia, tuberculosis, enteric fever, meningitis, etc.), any congenital anomaly or disorder (e.g. diagnosed inborn error of metabolism, congenital cardiac disease, seizure disorders, hypothyroidism, Down’s syndrome, etc.), a requirement of additional intravenous fluids after being provided with an IV for 4 h on admission if severely dehydrated, has documentation of taking antibiotics and/or antidiarrheal within the last 48 h before hospitalization. The study protocol is described in detail^60^.

**Figure 1:**
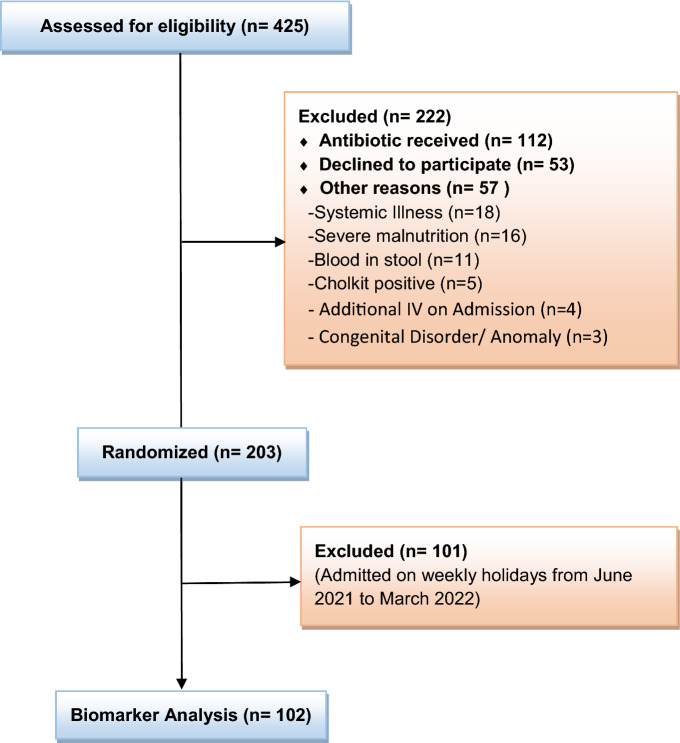
Study flow diagram.

For this study, the sample was restricted to 102 enrolled children based on the availability of tests done for biomarkers in the laboratory except for the weekly holidays from June 2021 to March 2022. To date, as there is no data available for this type of study, we estimated to collect first one-third of patients' EED biomarker data from the total enrolled children (n = 312) admitted in icddr,b Dhaka Hospital with dehydrating diarrhea based on our study budget and cost (Fig. 1, Supplementary file 1).

### Ethical consideration

The study was conducted following the Declaration of Helsinki of 1975, revised in 1983, and was approved by the Institutional Review Board (IRB) of icddr,b (protocol no PR-17028). The IRB of icddr,b comprises the Research Review Committee (RRC) and Ethical Review Committee (ERC). This study was registered as a clinical trial (www.ClinicalTrials.gov; NCT04677296. Registered on December 21, 2020). All methods were performed in accordance with the relevant guidelines and regulations. https://clinicaltrials.gov/ct2/show/NCT04677296?term=NCT04677296&draw=2&rank=1. Written informed consent was obtained from the parents or legal guardians of every child.

### Data collection

#### Case record form

Information on maternal and paternal education, household socioeconomic and demographic characteristics, infant and young child feeding (IYCF) practices (breastfeeding, and formula feeding), and vaccination were collected via a structured interview at the time of enrollment. A thorough clinical history and physical examination were done, and body weight and height were measured. For children, weight-for-age (WAZ), length-for-age (LAZ), and weight-for-length (WHZ) Z-scores were calculated according to the WHO growth standards at enrollment^63^.

### Variable of interest

Based on a comprehensive literature review, previous descriptive studies, and the availability of data in our investigation, several variables were considered explanatory variables. The outcome variables for this study were the level of gut health biomarkers: citrulline and KT ratio among the children having dehydrating diarrhea.

#### Household data

During hospital visits, detailed household data were obtained. The household demographic variables included type of floor (cemented or non-cemented), handwashing practices (before nursing or preparing baby food; after cleaning a child), access and the main source of drinking water (tube well water and non-tube well water), water treatment method (boiled or not), sanitation facilities (improved toilet facility for disposal of human fecal waste available or not), and the use of handwashing materials (water with soap or without soap). Parenteral education (illiterate or below primary level or primary and above), household size (number of children < 5 years of age), family income.

#### Feeding history

Breastfeeding referred to the breastfed baby or not. And whether the child was offered formula milk or not.

#### Vomiting and fever

Many of the components, such as vomiting (≥ 3 times/ day), and fever on admission (measured at least 38 degrees Celsius) were assessed.

#### Vaccination status

Defined as not vaccinated/vaccinated (age appropriate)/partially vaccinated, which can only be retrospectively assessed.

#### Measurement of stool output and hospital outcome

Stool output was measured from 4 h up to 120 h after randomization. We analyzed the data on total stool output (gram) till caseation of diarrhea (passage of soft or formed stool/no stool for 12 h) in the hospital, total duration of hospital stays in hours, and outcome of the child: usual discharge/treatment failure/DORB (Discharge on risk bond)/ dropout.

#### Stool sample collection and fecal microbiology

A single, fresh, stool specimen was collected from all enrolled patients at the time of enrollment, and immediately Cholkit test^64^ was done to exclude *Vibrio Cholerae*. Then submitted immediately to the clinical microbiology laboratory in Dhaka Hospital. All stool samples were routinely screened for common enteric pathogens, including bacterial pathogens (*Salmonella, Shigella, Vibrio cholerae*, *Campylobacter, Aeromonas* spp. and (enterotoxigenic, enteropathogenic, and enteroaggregative) *Escherichia coli*), rotaviruses antigen, and protozoa (*Giardia intestinalis, Entamoeba histolytica,* and *Cryptosporidium* spp.) have been identified by standard laboratory methods referred elsewhere^65,66^. Susceptibility to antimicrobials was determined by the standard disc diffusion method on Muller–Hinton agar with commercial discs (BD, Becton, Dickinson, and Company, Franklin Lakes, NJ), and the results were reported as S, I, R (sensitive, intermediate, and resistant) by a method based on the cutoff of the zone size for different antibiotics according to the latest available Clinical and Laboratory Standards Institute guidelines^67^.

#### Baseline blood biochemistry

Around 5 ml of venous blood samples were collected at enrolment and 24 h after the enrollment to determine serum electrolytes and blood glucose. We also performed a complete blood count, and serum creatinine, for all 102 patients on admission following standard microbiological laboratory procedures in the Dhaka Hospital, icddr,b^68^.

### Gut health biomarkers

On admission and 24 h after enrolment of the 102 children, plasma CIT and KT ratios were measured to assess the absorptive functions of the intestine and gut inflammation. The tests were done at the icddr,b laboratory, using the serum collected for routine blood testing, therefore no extra amount of blood or venipuncture was required. To separate the plasma, blood samples were centrifuged at 4000 rotations per minute (rpm) for 10 min. Quantitative analyses of plasma CIT concentrations and KT ratios are performed at icddr,b using the Enzyme-linked immunosorbent assay (ELISA) method.

### Determination of citrulline and K/T ratio with enzyme-linked immunosorbent assay (ELISA)

Citrulline, tryptophan (Immusmol, Bordeaux, France), and kynurenine (Immusmol, Bordeaux, France) levels were determined using commercially available kits following the manufacturer's instructions. The measurement ranges for kynurenine and tryptophan were from 0.5 to 50 μmol/L and 15 to 150 μmol/L respectively. All samples yielded measurable results and are included in the data presented. The KT ratio was obtained by dividing the plasma concentration of KYN (μmol/L) by the TRP concentration (μmol/L) and multiplying the quotient by 1000^16,17^. The methods to determine CIT and KT ratios are described in detail elsewhere^69,70^.

### Statistical analysis

We reported the child, parental, and household-level characteristics by using mean and standard deviation (SD) for continuous variables and frequency as a percentage for categorical variables to summarize the data. Raw data of EED biomarkers were subsequently normalized by log transformation. To examine the relations between biomarkers of EED at baseline and associated predictors, potential factors associated with citrulline, kynurenine, and tryptophan concentrations and the KT ratio were examined with linear regression; analyses were adjusted for age, breastfeeding status, wasting, lymphocyte count, maternal education, WASH and co-pathogen (EAEC and rotavirus) isolated from stool sample at enrollment; dependent variables were the EED biomarkers. In addition, to find the association between hospital outcome with EED biomarkers among diarrheal children we used a linear regression model where the outcome variables were duration of hospitalization (hours) till diarrhea ceased and total stool output during hospitalization in gram. Minimally adjusted separate models controlled for child age were used during the analysis (due to the small sample size, n = 102). To detect multicollinearity, the variance inflation factor (VIF) was calculated, and no variable producing a VIF value > 5 was found in the final model. The criterion of significance was set at 0.05, and 95% confidence intervals were calculated to determine the directions and strength of the effects. All data were analyzed using version 15.0 IC of STATA (College Station, TX, USA: Stata Corp LLC).

### Informed consent

Written informed consent was obtained from all the participants involved in this study.

## Supplementary Information

Supplementary Information.

## Data Availability

The dataset analyzed during the current study are not publicly available due to the data of this manuscript has been obtained from a clinical trial with a huge data set that deals with several objectives. The submitted manuscript deals with one of the objectives of that data set. This data set contains some personal information of the study patients (such as name, date of birth, admission date, month, and area of residence). However, during taking the consent from the patients, it has been ensured that their personal information of them will not be disclosed, but, the study results will be published. Thus, the availability of this whole data set in the manuscript, the supplemental files, or a public repository will open all the personal information of the patients that should not be disclosed; additionally, this will disclose other important information that is yet to be published. Thus, the policy of our center (icddr,b) is that we should not make the availability of the whole data set in the manuscript, the supplemental files, or a public repository. However, part of the data set related to this manuscript is available upon request and readers may contact Ms. Armana Ahmed (aahmed@icddrb.org) of the Research Administration & Strategy of icddr,b to request the data (http://www.icddrb.org/).
